# 4-Bromo­anilinium hydrogen phthalate

**DOI:** 10.1107/S1600536811017727

**Published:** 2011-05-14

**Authors:** Zu Pei Liang

**Affiliations:** a Department of Chemistry and Chemical Engineering, Weifang University, Weifang 261061, People’s Republic of China

## Abstract

In the anion of the title compound, C_6_H_7_BrN^+^·C_8_H_5_O_4_^−^, the dihedral angles formed by the benzene ring and the mean planes of the –COOH and –COO^−^ groups are 20.6 (3) and 83.2 (3)°, respectively. In the crystal, inter­molecular N—H⋯O and O—H⋯O hydrogen bonds connect the cations and anions, forming a two-dimensional network parallel to (001).

## Related literature

For applications of phthalimides and *N*-substituted phthalimides, see: Lima *et al.* (2002 ▶). For the crystal structures of 4-chloro­anilinium, 2-hy­droxy­anilinium and 3-hy­droxy­anilinium hydrogen phthalates, see: Jagan & Sivakumar (2009 ▶).
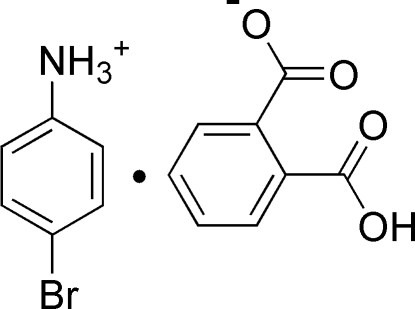


## Experimental

### 

#### Crystal data

C_6_H_7_BrN^+^·C_8_H_5_O_4_^−^
*M* *_r_* = 338.16
Monoclinic, 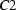

*a* = 13.0890 (14) Å
*b* = 7.6670 (7) Å
*c* = 14.6900 (14) Å
β = 106.671 (1)°
*V* = 1412.2 (2) Å^3^
*Z* = 4
Mo *K*α radiation
μ = 2.92 mm^−1^
*T* = 298 K
0.41 × 0.37 × 0.18 mm

#### Data collection

Bruker SMART CCD diffractometer
Absorption correction: multi-scan (*SADABS*; Bruker, 1997 ▶) *T*_min_ = 0.380, *T*_max_ = 0.621
3555 measured reflections
2364 independent reflections
1659 reflections with *I* > 2σ(*I*)
*R*_int_ = 0.045

#### Refinement

*R*[*F* ^2^ > 2σ(*F* ^2^)] = 0.054
*wR*(*F* ^2^) = 0.127
*S* = 0.94
2364 reflections
186 parameters
1 restraint
H atoms treated by a mixture of independent and constrained refinement
Δρ_max_ = 0.82 e Å^−3^
Δρ_min_ = −0.51 e Å^−3^
Absolute structure: Flack (1983 ▶), 1027 Friedel pairs
Flack parameter: 0.012 (16)

### 

Data collection: *SMART* (Bruker, 1997 ▶); cell refinement: *SAINT* (Bruker, 1997 ▶); data reduction: *SAINT*; program(s) used to solve structure: *SHELXS97* (Sheldrick, 2008 ▶); program(s) used to refine structure: *SHELXL97* (Sheldrick, 2008 ▶); molecular graphics: *SHELXTL* (Sheldrick, 2008 ▶) and *PLATON* (Spek, 2009 ▶); software used to prepare material for publication: *SHELXTL*.

## Supplementary Material

Crystal structure: contains datablocks global, I. DOI: 10.1107/S1600536811017727/lh5245sup1.cif

Structure factors: contains datablocks I. DOI: 10.1107/S1600536811017727/lh5245Isup2.hkl

Supplementary material file. DOI: 10.1107/S1600536811017727/lh5245Isup3.cml

Additional supplementary materials:  crystallographic information; 3D view; checkCIF report

## Figures and Tables

**Table 1:** Hydrogen-bond geometry (Å, °)

*D*—H⋯*A*	*D*—H	H⋯*A*	*D*⋯*A*	*D*—H⋯*A*
O2—H2⋯O3^i^	0.80 (6)	1.76 (6)	2.518 (6)	158 (7)
N1—H1*C*⋯O4	0.89	2.37	2.962 (7)	125
N1—H1*C*⋯O1^ii^	0.89	2.13	2.916 (7)	147
N1—H1*B*⋯O4^iii^	0.89	1.96	2.804 (7)	159
N1—H1*A*⋯O3^iv^	0.89	1.96	2.828 (6)	164

Symmetry codes: (i) 
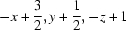
; (ii) 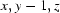; (iii) 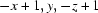; (iv) 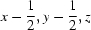.

**Fig. 1.**
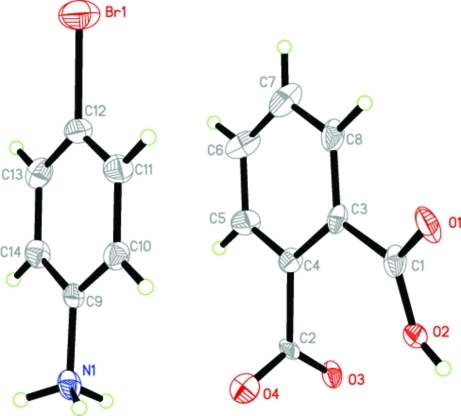
The asymmetric unit of (I), drawn with 30% probability ellipsoids.

**Fig. 2.**
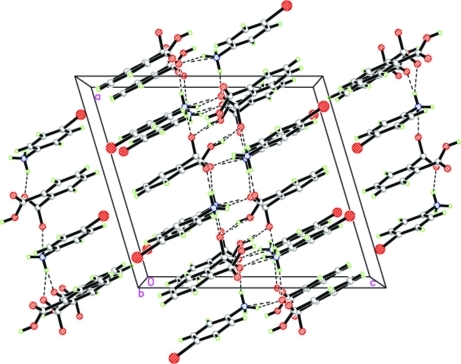
Part of the crystal structure of the title compound with hydrogen bonds shown as dashed lines.

## supplementary crystallographic information

### Comment

Phthalimides and N-substituted phthalimides are animportant class of compounds because of their interesting biological activities (Lima *et al.*, 2002). 4-Bromoanilinium hydrogen phthalate is an intermediate in the preparation of N-substituted phthalimides. The crystal structures of 4-chloroanilinium, 2-hydroxyanilinium and 3-hydroxyanilinium hydrogen phthalates have already been reported (Jagan & Sivakumar, 2009). In this paper, the structure of the title compound is reported. The asymmetric unit of the title compound (I) contains one 4-bromoanilinium cation and one hydrogen phthalate anion (Fig. 1). The dihedral angles formed by the benzene ring and the mean planes of the —COOH and —COO^-^ groups are 20.6 (3) and 83.2 (3) °, respectively. In the crystal, intermolecular N—H···O and O—H···O hydrogen bonds connect cations and anions to form a two-dimensional network parallel to (001) (Fig. 2).

### Experimental

A mixture of phthalic anhydride (1.52 g, 0.01 mol) and 4-bromoaniline (1.72 g, 0.01 mol) in 20 ml ethanol(95%) solution was refluxed for 0.5 h. The solution was kept at room temperature for 7 d. Natural evaporation gave colourless single crystals of the title compound, suitable for X-ray analysis.

### Refinement

H atoms bonded to C and N were initially located in difference maps and then refined in a riding-model approximation with C—H = 0.93 Å and N—H = 0.89 Å and with *U*_iso_(H) = 1.2*U*_eq_(C) or 1.5*U*_eq_(N). The H atom bonded to O was refined independently with an isotropic displacment parameter.

### Crystal data

**Table:**

C_6_H_7_BrN^+^·C_8_H_5_O_4_^−^	*F*(000) = 680
*M**_r_* = 338.16	*D*_x_ = 1.590 Mg m^−^^3^
Monoclinic, *C*2	Mo *K*α radiation, λ = 0.71073 Å
Hall symbol: C 2y	Cell parameters from 1173 reflections
*a* = 13.0890 (14) Å	θ = 2.9–20.8°
*b* = 7.6670 (7) Å	µ = 2.92 mm^−^^1^
*c* = 14.6900 (14) Å	*T* = 298 K
β = 106.671 (1)°	Block, colorless
*V* = 1412.2 (2) Å^3^	0.41 × 0.37 × 0.18 mm
*Z* = 4	

### Data collection

**Table:**

Bruker SMART CCD diffractometer	2364 independent reflections
Radiation source: fine-focus sealed tube	1659 reflections with *I* > 2σ(*I*)
graphite	*R*_int_ = 0.045
φ and ω scans	θ_max_ = 25.0°, θ_min_ = 2.9°
Absorption correction: multi-scan (*SADABS*; Bruker, 1997)	*h* = −13→15
*T*_min_ = 0.380, *T*_max_ = 0.621	*k* = −8→9
3555 measured reflections	*l* = −17→13

### Refinement

**Table:**

Refinement on *F*^2^	Secondary atom site location: difference Fourier map
Least-squares matrix: full	Hydrogen site location: inferred from neighbouring sites
*R*[*F*^2^ > 2σ(*F*^2^)] = 0.054	H atoms treated by a mixture of independent and constrained refinement
*wR*(*F*^2^) = 0.127	*w* = 1/[σ^2^(*F*_o_^2^) + (0.0642*P*)^2^] where *P* = (*F*_o_^2^ + 2*F*_c_^2^)/3
*S* = 0.94	(Δ/σ)_max_ < 0.001
2364 reflections	Δρ_max_ = 0.82 e Å^−^^3^
186 parameters	Δρ_min_ = −0.51 e Å^−^^3^
1 restraint	Absolute structure: Flack (1983), 1027 Friedel pairs
Primary atom site location: structure-invariant direct methods	Flack parameter: 0.012 (16)

### Special details

**Table:**

Geometry. All e.s.d.'s (except the e.s.d. in the dihedral angle between two l.s. planes) are estimated using the full covariance matrix. The cell e.s.d.'s are taken into account individually in the estimation of e.s.d.'s in distances, angles and torsion angles; correlations between e.s.d.'s in cell parameters are only used when they are defined by crystal symmetry. An approximate (isotropic) treatment of cell e.s.d.'s is used for estimating e.s.d.'s involving l.s. planes.
Refinement. Refinement of *F*^2^ against ALL reflections. The weighted *R*-factor *wR* and goodness of fit *S* are based on *F*^2^, conventional *R*-factors *R* are based on *F*, with *F* set to zero for negative *F*^2^. The threshold expression of *F*^2^ > σ(*F*^2^) is used only for calculating *R*-factors(gt) *etc*. and is not relevant to the choice of reflections for refinement. *R*-factors based on *F*^2^ are statistically about twice as large as those based on *F*, and *R*- factors based on ALL data will be even larger.

### Fractional atomic coordinates and isotropic or equivalent isotropic displacement parameters (Å^2^)

**Table:**

	*x*	*y*	*z*	*U*_iso_*/*U*_eq_	
Br1	0.16160 (6)	0.43398 (15)	0.05674 (5)	0.0677 (3)	
N1	0.3761 (4)	−0.0249 (6)	0.4004 (3)	0.0329 (13)	
H1A	0.3407	−0.1253	0.3952	0.049*	
H1B	0.3731	0.0303	0.4528	0.049*	
H1C	0.4438	−0.0462	0.4035	0.049*	
O1	0.5651 (4)	0.7623 (6)	0.4163 (4)	0.0553 (16)	
O2	0.6767 (3)	0.5378 (6)	0.4617 (4)	0.0377 (12)	
H2	0.699 (4)	0.569 (9)	0.516 (4)	0.03 (2)*	
O3	0.7303 (3)	0.1975 (5)	0.3875 (3)	0.0324 (10)	
O4	0.5788 (4)	0.1691 (7)	0.4309 (4)	0.0385 (12)	
C1	0.5990 (5)	0.6178 (9)	0.4009 (6)	0.0345 (18)	
C2	0.6314 (5)	0.2287 (7)	0.3804 (5)	0.0255 (15)	
C3	0.5581 (5)	0.5282 (9)	0.3075 (6)	0.0307 (17)	
C4	0.5732 (5)	0.3475 (9)	0.2962 (5)	0.0297 (17)	
C5	0.5343 (5)	0.2691 (11)	0.2082 (5)	0.044 (2)	
H5	0.5446	0.1504	0.2013	0.053*	
C6	0.4791 (6)	0.3685 (13)	0.1293 (6)	0.056 (3)	
H6	0.4520	0.3151	0.0704	0.067*	
C7	0.4648 (6)	0.5461 (13)	0.1385 (7)	0.056 (2)	
H7	0.4297	0.6127	0.0859	0.067*	
C8	0.5031 (5)	0.6231 (10)	0.2269 (6)	0.043 (2)	
H8	0.4920	0.7418	0.2329	0.052*	
C9	0.3276 (4)	0.0844 (8)	0.3171 (4)	0.0305 (15)	
C10	0.3175 (5)	0.2651 (8)	0.3301 (5)	0.0342 (16)	
H10	0.3433	0.3149	0.3901	0.041*	
C11	0.2688 (4)	0.3668 (9)	0.2525 (5)	0.0403 (17)	
H11	0.2614	0.4862	0.2598	0.048*	
C12	0.2314 (5)	0.2920 (9)	0.1649 (5)	0.0378 (17)	
C13	0.2417 (5)	0.1129 (9)	0.1509 (5)	0.0425 (17)	
H13	0.2166	0.0638	0.0908	0.051*	
C14	0.2908 (4)	0.0107 (8)	0.2296 (5)	0.0356 (16)	
H14	0.2983	−0.1086	0.2223	0.043*	

### Atomic displacement parameters (Å^2^)

**Table:**

	*U*^11^	*U*^22^	*U*^33^	*U*^12^	*U*^13^	*U*^23^
Br1	0.0717 (5)	0.0667 (6)	0.0567 (5)	0.0124 (6)	0.0056 (3)	0.0159 (6)
N1	0.033 (2)	0.020 (3)	0.047 (3)	−0.002 (2)	0.013 (2)	−0.003 (2)
O1	0.052 (3)	0.027 (3)	0.082 (4)	0.015 (2)	0.011 (3)	−0.012 (3)
O2	0.035 (2)	0.024 (2)	0.045 (3)	0.0035 (19)	−0.002 (2)	−0.009 (2)
O3	0.025 (2)	0.021 (2)	0.050 (3)	0.0016 (17)	0.0098 (19)	0.001 (2)
O4	0.033 (2)	0.040 (3)	0.043 (3)	−0.007 (2)	0.013 (2)	0.004 (2)
C1	0.019 (3)	0.030 (4)	0.055 (5)	0.002 (3)	0.011 (3)	0.005 (3)
C2	0.029 (4)	0.011 (3)	0.036 (4)	−0.002 (2)	0.008 (3)	−0.007 (2)
C3	0.016 (3)	0.036 (4)	0.040 (4)	0.004 (3)	0.007 (3)	0.007 (3)
C4	0.032 (4)	0.022 (4)	0.034 (5)	−0.004 (3)	0.006 (3)	0.007 (3)
C5	0.043 (4)	0.043 (5)	0.044 (5)	−0.007 (3)	0.009 (4)	−0.004 (4)
C6	0.043 (4)	0.074 (7)	0.044 (5)	−0.013 (4)	0.001 (4)	−0.002 (4)
C7	0.039 (4)	0.066 (7)	0.054 (6)	−0.008 (4)	−0.001 (4)	0.017 (5)
C8	0.033 (4)	0.027 (4)	0.063 (6)	0.003 (3)	0.003 (4)	0.014 (4)
C9	0.021 (3)	0.035 (4)	0.037 (4)	−0.003 (3)	0.010 (3)	0.008 (3)
C10	0.032 (3)	0.030 (4)	0.038 (4)	−0.002 (3)	0.005 (3)	−0.004 (3)
C11	0.036 (3)	0.034 (4)	0.050 (5)	0.005 (3)	0.012 (3)	0.005 (3)
C12	0.033 (3)	0.041 (5)	0.038 (4)	0.003 (3)	0.007 (3)	0.008 (3)
C13	0.036 (4)	0.052 (5)	0.038 (4)	−0.004 (3)	0.008 (3)	−0.007 (3)
C14	0.037 (3)	0.024 (4)	0.045 (4)	0.000 (3)	0.012 (3)	−0.006 (3)

### Geometric parameters (Å, °)

**Table:**

Br1—C12	1.927 (6)	C5—H5	0.9300
N1—C9	1.468 (8)	C6—C7	1.386 (12)
N1—H1A	0.8900	C6—H6	0.9300
N1—H1B	0.8900	C7—C8	1.382 (11)
N1—H1C	0.8900	C7—H7	0.9300
O1—C1	1.238 (8)	C8—H8	0.9300
O2—C1	1.299 (8)	C9—C14	1.359 (8)
O2—H2	0.80 (6)	C9—C10	1.410 (9)
O3—C2	1.292 (7)	C10—C11	1.377 (9)
O4—C2	1.236 (8)	C10—H10	0.9300
C1—C3	1.490 (11)	C11—C12	1.365 (9)
C2—C4	1.548 (9)	C11—H11	0.9300
C3—C8	1.400 (10)	C12—C13	1.400 (10)
C3—C4	1.415 (8)	C13—C14	1.392 (9)
C4—C5	1.385 (11)	C13—H13	0.9300
C5—C6	1.402 (12)	C14—H14	0.9300
			
C9—N1—H1A	109.5	C8—C7—C6	119.2 (9)
C9—N1—H1B	109.5	C8—C7—H7	120.4
H1A—N1—H1B	109.5	C6—C7—H7	120.4
C9—N1—H1C	109.5	C7—C8—C3	122.2 (8)
H1A—N1—H1C	109.5	C7—C8—H8	118.9
H1B—N1—H1C	109.5	C3—C8—H8	118.9
C1—O2—H2	122 (5)	C14—C9—C10	121.0 (6)
O1—C1—O2	123.2 (7)	C14—C9—N1	120.1 (6)
O1—C1—C3	121.9 (7)	C10—C9—N1	118.9 (6)
O2—C1—C3	114.8 (6)	C11—C10—C9	118.9 (6)
O4—C2—O3	127.0 (6)	C11—C10—H10	120.5
O4—C2—C4	117.8 (6)	C9—C10—H10	120.5
O3—C2—C4	115.2 (5)	C12—C11—C10	119.9 (6)
C8—C3—C4	117.7 (8)	C12—C11—H11	120.1
C8—C3—C1	120.1 (7)	C10—C11—H11	120.1
C4—C3—C1	122.2 (7)	C11—C12—C13	121.8 (6)
C5—C4—C3	120.5 (8)	C11—C12—Br1	119.8 (5)
C5—C4—C2	117.1 (6)	C13—C12—Br1	118.4 (5)
C3—C4—C2	122.4 (7)	C14—C13—C12	118.1 (6)
C4—C5—C6	120.0 (8)	C14—C13—H13	121.0
C4—C5—H5	120.0	C12—C13—H13	121.0
C6—C5—H5	120.0	C9—C14—C13	120.4 (6)
C7—C6—C5	120.3 (9)	C9—C14—H14	119.8
C7—C6—H6	119.8	C13—C14—H14	119.8
C5—C6—H6	119.8		
			
O1—C1—C3—C8	18.0 (10)	C5—C6—C7—C8	−1.6 (13)
O2—C1—C3—C8	−157.9 (6)	C6—C7—C8—C3	1.3 (12)
O1—C1—C3—C4	−162.5 (7)	C4—C3—C8—C7	−0.3 (10)
O2—C1—C3—C4	21.7 (9)	C1—C3—C8—C7	179.3 (7)
C8—C3—C4—C5	−0.2 (10)	C14—C9—C10—C11	0.4 (9)
C1—C3—C4—C5	−179.8 (6)	N1—C9—C10—C11	−177.8 (5)
C8—C3—C4—C2	−179.1 (5)	C9—C10—C11—C12	0.0 (9)
C1—C3—C4—C2	1.3 (10)	C10—C11—C12—C13	−0.5 (10)
O4—C2—C4—C5	−95.0 (7)	C10—C11—C12—Br1	179.2 (4)
O3—C2—C4—C5	82.4 (7)	C11—C12—C13—C14	0.7 (9)
O4—C2—C4—C3	83.9 (8)	Br1—C12—C13—C14	−179.0 (4)
O3—C2—C4—C3	−98.7 (7)	C10—C9—C14—C13	−0.2 (8)
C3—C4—C5—C6	−0.1 (11)	N1—C9—C14—C13	178.0 (5)
C2—C4—C5—C6	178.9 (6)	C12—C13—C14—C9	−0.3 (9)
C4—C5—C6—C7	1.0 (12)		

### Hydrogen-bond geometry (Å, °)

**Table:**

*D*—H···*A*	*D*—H	H···*A*	*D*···*A*	*D*—H···*A*
O2—H2···O3^i^	0.80 (6)	1.76 (6)	2.518 (6)	158 (7)
N1—H1C···O4	0.89	2.37	2.962 (7)	125
N1—H1C···O1^ii^	0.89	2.13	2.916 (7)	147
N1—H1B···O4^iii^	0.89	1.96	2.804 (7)	159
N1—H1A···O3^iv^	0.89	1.96	2.828 (6)	164

Symmetry codes: (i) −*x*+3/2, *y*+1/2, −*z*+1; (ii) *x*, *y*−1, *z*; (iii) −*x*+1, *y*, −*z*+1; (iv) *x*−1/2, *y*−1/2, *z*.

## References

[bb1] Bruker (1997). *SADABS*, *SMART* and *SAINT*, Bruker AXS Inc., Madison, Wisconsin, USA.

[bb2] Flack, H. D. (1983). *Acta Cryst.* A**39**, 876–881.

[bb3] Jagan, R. & Sivakumar, K. (2009). *Acta Cryst.* C**65**, o414–o418. 10.1107/S0108270109025724 19652327

[bb4] Lima, L. M., Castro, P., Machado, A. L., Frage, C. A. M., Lugniur, C., Moraes, V. L. G. & Barreiro, E. (2002). *J. Biol. Org. Med. Chem.* **10**, 3067–3073. 10.1016/s0968-0896(02)00152-9 12110331

[bb5] Sheldrick, G. M. (2008). *Acta Cryst.* A**64**, 112–122. 10.1107/S0108767307043930 18156677

[bb6] Spek, A. L. (2009). *Acta Cryst.* D**65**, 148–155. 10.1107/S090744490804362X PMC2631630 19171970

